# Point-of-care ultrasound evaluation of obstructive azoospermia in a patient with prior bilateral Cohen ureteral reimplantation: A case report

**DOI:** 10.1186/s12610-026-00321-5

**Published:** 2026-08-03

**Authors:** Shlomi Barak, Netanel Waldenberg, Guy Bar, Oshri Barel, Snir Dekalo, Atef Zeadna, Benzion Samueli, Guy Shrem

**Affiliations:** 1 Samson Assuta Ashdod University Hospital, Reproductive Services, Ashdod, Israel; 2 Faculty of Health Sciences, Ben-Gurion University of the Negev, Beer- Sheva, Israel; 3 Fertility and IVF Unit, Department of Obstetrics and Gynecology, Soroka University Medical Center, Be’er Sheva, Israel; 4 Urology department, Tel Aviv Sourasky Medical Center, Faculty of Medical health and sciences, Tel Aviv, Israel; 5 Department of Pathology, Soroka University Medical Center, Beer-Sheva, Israel; 6 The Azrieli Faculty of Medicine, Bar Ilan University, Safed, 1361101 Israel; 7 Fertility Clinic, Clalit Health Services, North district, Migdal-HaEmek, Israel

**Keywords:** Obstructive azoospermia, point-of-care ultrasound, male infertility, Cohen ureteral reimplantation, Azoospermie obstructive, Echographie au Point de Soins, Infertilité masculine, Réimplantation urétérale de Cohen

## Abstract

**Background:**

Obstructive azoospermia (OA) is a potentially treatable cause of male infertility. Point-of-care ultrasonography (POCUS) has emerged as a valuable adjunct in the evaluation of male infertility, enabling rapid identification of sonographic findings suggestive of reproductive tract obstruction. We describe the use of scrotal POCUS in the evaluation of OA in a man with a history of infantile bilateral Cohen cross-trigonal ureteral reimplantation for severe vesicoureteral reflux.

**Case presentation:**

A 32-year-old man presented with primary infertility and azoospermia, with normal semen volume and pH. His medical history was notable for severe grade V vesicoureteral reflux treated with bilateral Cohen cross-trigonal ureteral reimplantation at 4 months of age. Physical examination demonstrated bilaterally palpable vasa deferentia and normal testes. Hormonal evaluation and genetic testing were unremarkable. Scrotal POCUS revealed multiple cystic structures in both epididymides with otherwise normal-appearing testes, findings highly suggestive of OA. A normal hormonal profile further supported an obstructive etiology. In the absence of alternative etiologies, OA was considered the most likely diagnosis. The patient subsequently underwent successful testicular sperm aspiration (TESA), yielding abundant motile sperm for cryopreservation, with histopathologic confirmation of normal spermatogenesis.

**Conclusions:**

This case highlights the potential value of scrotal POCUS as a rapid bedside tool that can support the diagnosis of OA and facilitate timely clinical decision-making. In patients with a history of prior genitourinary surgery, characteristic sonographic findings may help guide management and expedite fertility treatment. Further studies are warranted to better define the role of POCUS in the diagnostic evaluation of male infertility.

## Résumé

**Contexte:**

L’azoospermie obstructive (AO) est une cause potentiellement traitable de l’infertilité masculine. L’échographie au point de soins (POCUS) s’est imposée comme un complément précieux dans l’évaluation de l’infertilité masculine, permettant d’identifier rapidement les résultats échographiques suggérant une obstruction du tractus reproducteur. Nous décrivons l’utilisation du POCUS scrotal dans l’évaluation d’une AO chez un homme ayant des antécédents infantiles de réimplantation urétérale bilatérale par la méthode trigonale croisée de Cohen pour un sévère reflux vésico-urétéral.

**Présentation du Cas:**

Un homme de 32 ans présentait une infertilité primaire et une azoospermie, avec un volume et un pH de sperme normaux. Ses antécédents médicaux présentaient notablement un reflux vésico-urétéral sévère de grade V traité par une réimplantation urétérale bilatérale croisée de Cohen à l’âge de 4 mois. L’examen physique a révélé un canal déférent palpable bilatéralement et des testicules normaux. L’évaluation hormonale et les tests génétiques étaient sans intérêt notable. Le POCUS scrotal a révélé de multiples structures kystiques dans les deux épididymides avec des testicules d’apparence normales, des résultats suggérant fortement une AO. Un profil hormonal normal a renforcé une étiologie obstructive. En l’absence d’étiologies alternatives, l’AO a été considérée comme le diagnostic le plus probable. Le patient a ensuite subi une aspiration testiculaire réussie, avec une abondance de spermatozoïdes mobiles pour la cryopréservation, et la confirmation histopathologique d’une spermatogenèse normale.

**Conclusion:**

Ce cas met en lumière la valeur potentielle du POCUS scrotal comme outil rapide au lit du patient pouvant soutenir le diagnostic d’AO et faciliter la prise de décision clinique en temps voulu. Chez les patients avec antécédents de chirurgie génito-urinaire antérieure, des résultats échographiques caractéristiques peuvent aider à orienter la prise en charge et à accélérer le traitement de la fertilité. Des études supplémentaires sont nécessaires pour mieux définir le rôle du POCUS dans l’évaluation diagnostique de l’infertilité masculine.

## Attestation statement

- The subjects in this trial have not concomitantly been involved in other randomized trials.
- Data regarding any of the subjects in the study has not been previously published unless specified.
- Data will be made available to the editors of the journal for review or query upon request pre and/or post publication.
- The appropriate CARE checklist for this study design was followed.

## Introduction

Azoospermia, defined as the complete absence of spermatozoa in the ejaculate, is identified in approximately 10%–15% of men undergoing fertility evaluation [1]. Distinguishing obstructive azoospermia (OA) from nonobstructive azoospermia (NOA) is of critical clinical importance because it directly influences patient counseling, management, and treatment options [2]. The evaluation of azoospermic men typically includes a detailed history, physical examination, semen analysis, hormonal assessment, genetic testing, and imaging studies [3]. According to AUA/ASRM guidelines, men with normal follicle-stimulating hormone (FSH) levels and normal testicular volume may be offered testicular biopsy to clarify the underlying etiology [4].

**Fig. 1:**
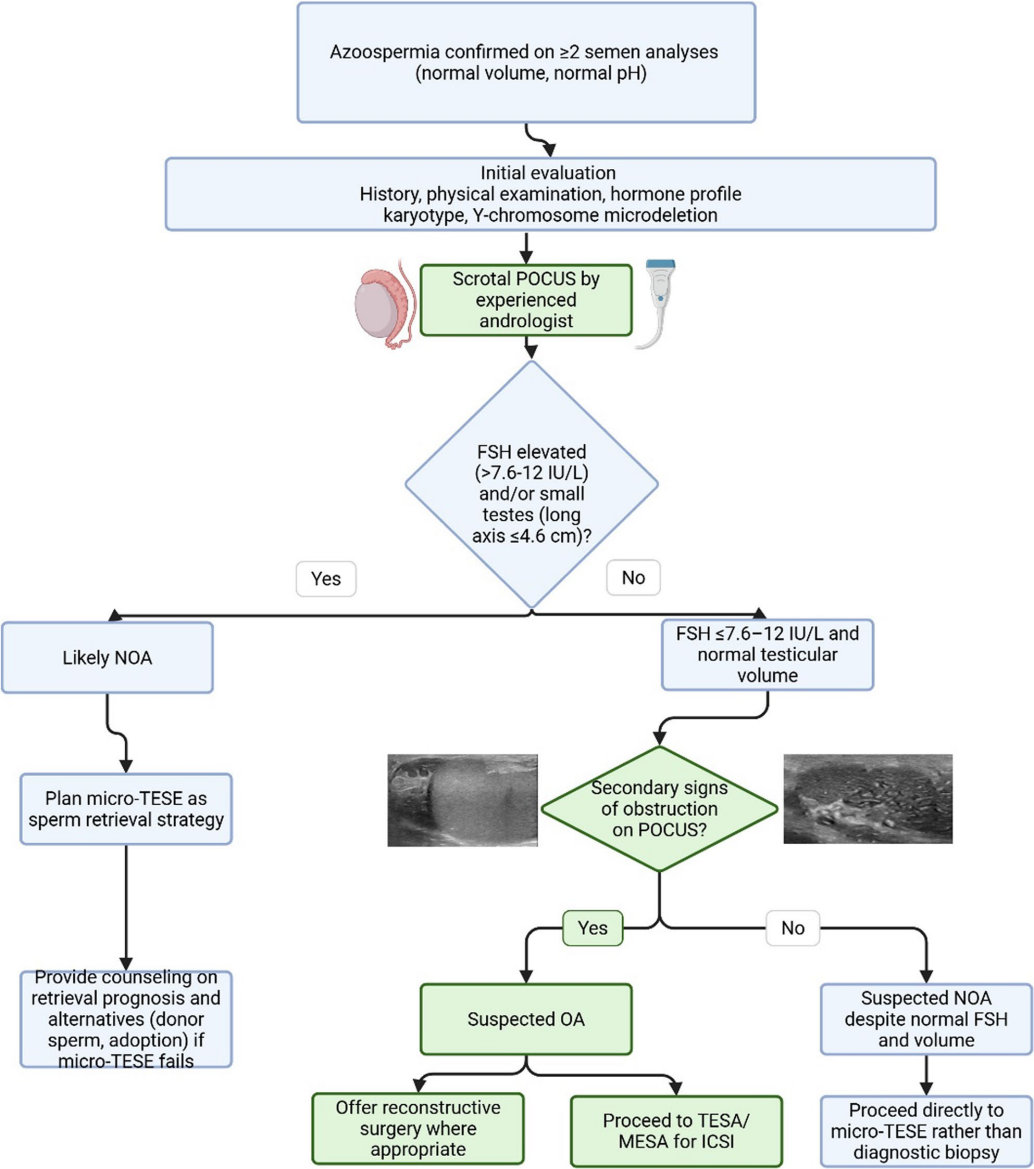
Proposed scrotal POCUS algorithm for differentiating obstructive azoospermia (OA) from non-obstructive azoospermia (NOA). Schematic representation of the diagnostic approach used during the initial evaluation of azoospermic men. After confirmation of azoospermia and completion of the standard clinical, hormonal, and genetic workup, scrotal point-of-care ultrasonography (POCUS) is used to assess testicular volume and identify secondary sonographic signs of obstruction. The algorithm assists in distinguishing OA from NOA and in selecting the most appropriate management strategy

Point-of-care ultrasonography (POCUS) is increasingly recognized as a valuable bedside diagnostic tool across multiple medical disciplines [5] (Fig. 1). By integrating sonographic findings with the clinical evaluation in real time, POCUS can support rapid clinical decision-making. Handheld ultrasound devices are now widely available, and studies have demonstrated that appropriately trained non-radiologists can perform focused ultrasound examinations reliably [6, 7]. In the evaluation of azoospermia, scrotal POCUS may help identify sonographic features suggestive of reproductive tract obstruction and contribute to the differentiation of OA from NOA [8].

As part of their infertility evaluation, it is crucial to consider the surgical history of patients, as may have unrecognized long-term implications.

Primary vesicoureteral reflux (VUR) is a common congenital urinary tract abnormality characterized by retrograde urine flow from the bladder into the ureter due to dysfunction of the ureterovesical junction [9–11]. Surgical correction is highly effective and is associated with excellent long-term outcomes [12]. Cohen cross-trigonal ureteral reimplantation remains one of the most widely performed intravesical techniques for the treatment of VUR [13].

We present the case of a man with a history of bilateral Cohen ureteral reimplantation during infancy who was evaluated for azoospermia in adulthood. Scrotal POCUS identified findings highly suggestive of reproductive tract obstruction and contributed to the clinical assessment and management of the patient. To our knowledge, this is the first reported case describing the use of point-of-care scrotal ultrasound in the evaluation of obstructive azoospermia in a patient with a history of infantile bilateral Cohen ureteral reimplantation. This report highlights the potential role of POCUS in the assessment of azoospermic men and emphasizes the importance of considering prior pediatric urologic surgery during infertility evaluation.

### Case presentation

A 32-year-old man was referred to our andrology outpatient clinic after experiencing one year of primary infertility. He had no significant medical illnesses in adulthood and was not taking any medications. However, his pediatric history was notable for severe grade V primary vesicoureteral reflux diagnosed in early infancy. At 4 months of age, he underwent bilateral Cohen cross-trigonal ureteral reimplantation for severe grade V vesicoureteral reflux. According to the available medical records, the surgery and recovery were uneventful, and the patient did not experience any urinary tract infections or kidney problems thereafter. Detailed documentation regarding the specific indications and timing of surgical intervention was not available for review. He had achieved normal developmental milestones and had no issues with puberty or sexual function.

On examination, the patient was well virilized, with normal secondary sexual characteristics. Both testes were palpable in the scrotum, of normal size (~ 18 mL bilaterally) and consistency. No palpable masses or varicocele were observed. The epididymides were not clearly distinguishable on physical examination. The vas deferens was palpated bilaterally. There was no history of scrotal trauma, infection (e.g., epididymo-orchitis), or inguinal surgery. The patient’s partner was evaluated, and no infertility factors were identified.

Three consecutive semen analyses confirmed a diagnosis of azoospermia. The seminal fluid volume was 2.5-4 mL with normal pH (7.8). The patient’s endocrine workup was unremarkable: serum follicle-stimulating hormone (FSH) was 2.4 mIU/mL, luteinizing hormone was 1.9 mIU/mL, and total testosterone 17.5 nmol/L. Genetic evaluation, including cystic fibrosis mutation screening, karyotype analysis, and Y chromosome microdeletion testing, was performed according to our routine institutional protocol for azoospermia evaluation and yielded negative results.

**Fig. 2:**
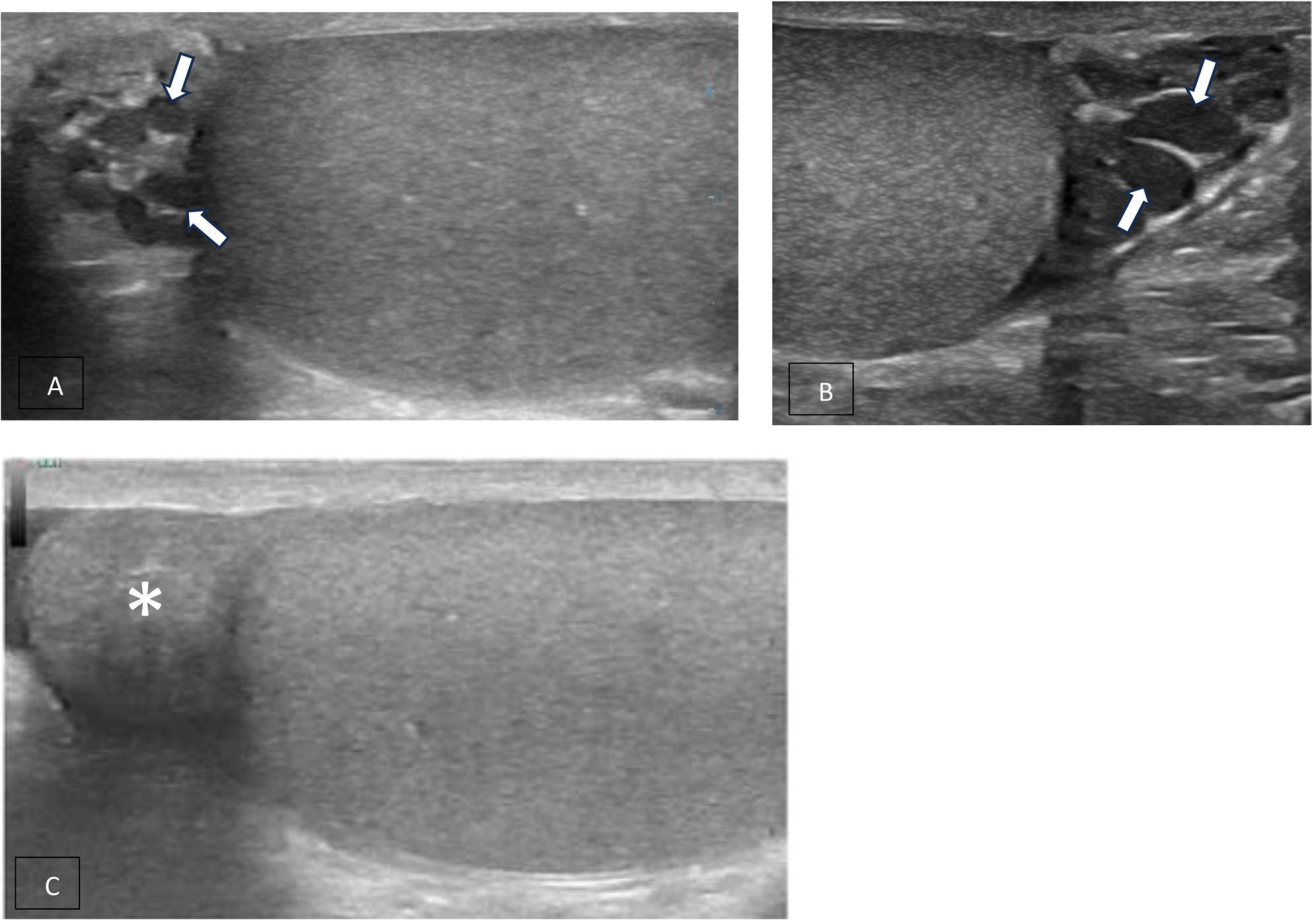
Scrotal point-of-care ultrasonography findings suggestive of obstructive azoospermia. (**A**, **B**) Scrotal POCUS images demonstrating multiple anechoic epididymal cysts (arrows) within the epididymal head, interpreted as secondary sonographic signs suggestive of obstruction. (**C**) Normal appearance of the epididymal head (asterisk) shown for comparison. These findings were interpreted in conjunction with the clinical history, physical examination, and hormonal profile

A high-frequency linear transducer was used by a clinician (andrologist) to perform a focused POCUS examination of the testes and epididymides. Both testes appeared unremarkable with no hypoechogenic findings. The epididymides were bilaterally enlarged with numerous small (< 5 mm) epididymal cysts scattered within the head and tail (Fig. 2). No varicocele was observed. There was no hydrocele. These sonographic findings suggested that the patient’s azoospermia was likely obstructive. A formal scrotal ultrasound examination had previously been reported as normal, without documented epididymal abnormalities.

**Fig. 3:**
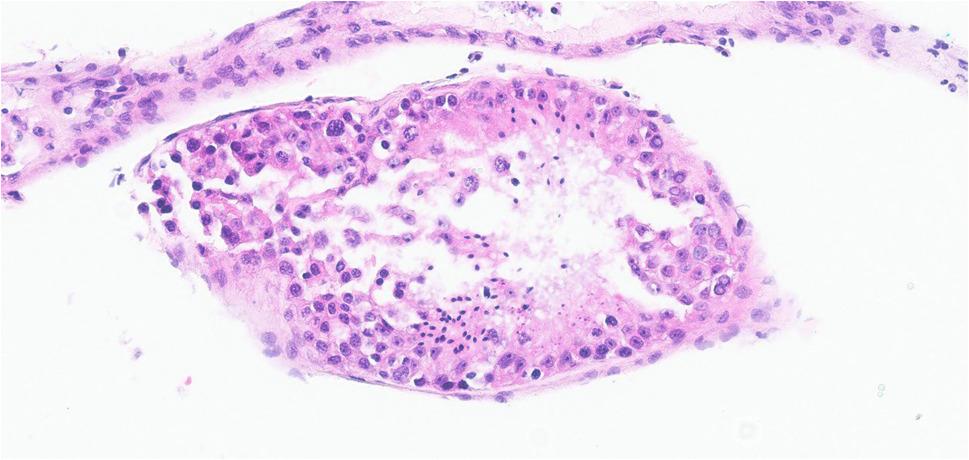
Histopathologic findings demonstrating preserved spermatogenesis following testicular sperm aspiration (TESA). Histologic examination of the testicular biopsy specimen demonstrated fewer than 25 seminiferous tubules, representing a relatively small but adequate sample for evaluation [15]. Spermatozoa were identified in several tubules, confirming preserved spermatogenesis and supporting the diagnosis of obstructive azoospermia. Hematoxylin and eosin (H&E) stain; original magnification ×400

Based on the POCUS findings, along with clinical examination, hormone profile, and genetic assessment, the working diagnosis was OA, and the patient was counseled accordingly. Alternative causes of obstructive azoospermia, including prior inguinal or scrotal surgery with vasal injury, postinfectious or inflammatory epididymal obstruction, congenital vasal anomalies, distal seminal tract obstruction, trauma-related obstruction, and idiopathic obstruction, were considered but were not supported by the patient’s clinical history, physical examination, genetic evaluation, or sonographic findings. In this context, the patient’s history of bilateral Cohen ureteral reimplantation was considered a potential contributing factor and was interpreted together with the overall clinical findings. The patient was counseled regarding available management options, including sperm retrieval with assisted reproductive technologies or referral for further evaluation of potential reconstructive management. He elected to proceed directly with sperm retrieval for IVF-ICSI and did not pursue further consultation regarding reconstructive surgery. Unilateral testicular sperm aspiration (TESA) was performed, yielding abundant motile sperm (Fig. 3). The TESA yielded abundant motile sperm, strongly supporting the diagnosis of OA, and provided sperm that was cryopreserved (20 vials) for future in vitro fertilization with intracytoplasmic sperm injection (IVF-ICSI). Histopathology confirmed normal spermatogenesis [14]. The patient and his partner were relieved to receive a clear diagnosis and plan.

The present study’s protocol was reviewed and approved by our Institutional Review Board (approval No. 2023001). Written informed consent was obtained from the patient for participation in this report and for publication of his clinical details. All potentially identifying information has been omitted to preserve patient confidentiality.

## Discussion

This case demonstrates how POCUS examination can significantly streamline the diagnostic process for infertile patients, particularly in distinguishing between OA and NOA. Typically, an azoospermic man may undergo numerous investigations before reaching a definitive diagnosis, including scrotal ultrasonography performed by a radiologist, transrectal ultrasound of the prostate/seminal vesicles, laboratory tests, and even a diagnostic testicular biopsy. In this instance, a focused POCUS examination conducted by the treating andrologist during the initial clinic visit immediately revealed sonographic features suggestive of obstruction, including multiple bilateral epididymal cysts, which were interpreted in conjunction with the clinical history, physical examination, and hormonal profile. This allowed us to strongly suspect obstructive azoospermia during the initial encounter and proceed efficiently with further management. This approach aligns with our recent study, which suggests that scrotal POCUS performed by a fertility specialist or andrologist may help differentiate OA from NOA and may reduce the need for empirical exploratory surgery in selected cases [8]. In our recent study, scrotal POCUS demonstrated high diagnostic performance in distinguishing OA from NOA in azoospermic patients with normal semen volume and pH, with a reported PPV of 96.4% and NPV of 100% [8]. An intriguing aspect of this case is the possible association between the patient’s obstructive azoospermia and his history of bilateral ureteral reimplantation during infancy, although a causal relationship cannot be established from a single case.

Cohen bilateral ureteral reimplantation is a widely used surgical technique for correcting vesicoureteral reflux (VUR). Although generally effective, it comes with important safety considerations and potential complications. This procedure involves intravesical reimplantation of the ureters, which can be performed through open surgery or minimally invasive methods such as laparoscopic or robotically assisted techniques. The Cohen technique necessitates careful dissection near the bladder trigone, where the vas deferens is in close proximity, increasing the risk of iatrogenic injury to this structure. Although direct data on vas deferens injury during Cohen reimplantation are scarce, pediatric and genitourinary surgery literature indicate that vas deferens injury is a recognized risk during pelvic procedures involving the ureter or spermatic cord [16]. Minimally invasive approaches using robotic assistance have been explored to reduce morbidity while maintaining its efficacy. Robotically assisted bilateral intravesical ureteral reimplantation has shown promising outcomes, with fewer intraoperative complications reported in small pediatric case series, although direct reports of vas deferens injuries are limited [17]. The precision of the robotic technique is believed to potentially reduce the risk of injury owing to enhanced visualization and delicate instrument control [18, 19]. Overall, avoiding vas deferens injury during Cohen bilateral reimplantation requires meticulous surgical technique, detailed anatomical knowledge, and careful dissection to preserve the reproductive structures. Despite these inherent risks, the procedure remains a standard and effective treatment for VUR, with well-documented outcomes. Continuous advancements in minimally invasive and robotic techniques aim to minimize surgical complications while preserving adjacent reproductive structures. Although such complications are uncommon, awareness of their potential long-term implications remains important when evaluating adult men presenting with infertility. The reported case underscores the critical need for adult healthcare providers to obtain a comprehensive pediatric history in men experiencing infertility. Awareness of the patient’s previous genitourinary surgery helped place the POCUS findings in the appropriate clinical context. In our practice, scrotal POCUS is routinely incorporated into the evaluation of azoospermic men; however, recognition of this surgical history increased our suspicion for a possible obstructive etiology and facilitated interpretation of the sonographic findings.

Clinically, this report provides several important clinical insights. First, POCUS serves as a valuable tool for assessing male infertility by offering real-time insights that enhance laboratory tests and physical examinations. With proper training, andrologists and urologists can use handheld ultrasound devices to swiftly identify signs of obstruction or other conditions, such as varicocele, during the initial visit, thereby enhancing diagnostic efficiency. Incorporating POCUS into clinical practice could shorten the time between infertility diagnosis and definitive treatment, as illustrated in this case.

Second, even a seemingly distant medical history can be pertinent; men with a history of significant childhood genitourinary surgeries, such as ureteral reimplantation, may warrant assessment for potential sequelae affecting fertility. These patients may benefit from early imaging evaluation of the reproductive tract. Although pediatric surgical techniques continue to advance, and long-term outcomes are generally positive, maintaining a high level of suspicion for atypical late effects, albeit rare, ensures that nothing is overlooked. In our case, consideration of the patient’s prior Cohen procedure contributed to the overall clinical assessment and supported the interpretation of the obstructive findings. Lastly, this case contributes to the growing evidence supporting multidisciplinary awareness: pediatric urologists might be aware that, in extremely rare circumstances, a history of complex childhood genitourinary surgery may become relevant during future infertility evaluations. Conversely, adult practitioners should be confident in consulting pediatric surgical records or colleagues when encountering unusual infertility cases.

### Limitations

This report describes a single case, which inherently limits the generalizability of our findings. While the clinical findings support a possible association, a direct causal relationship cannot be established from a single case report. Furthermore, the exact mechanism of obstruction (e.g., vas deferens injury) could not be precisely determined without further invasive diagnostics, which were not pursued given the successful sperm retrieval.

## Conclusion

We present a unique case of obstructive azoospermia in a 32-year-old man with a history of bilateral Cohen ureteral reimplantation performed during infancy for vesicoureteral reflux. Although a causal relationship cannot be established from a single case, this report raises awareness of a possible association that may be relevant during infertility evaluation.

Point-of-care scrotal ultrasonography provided important sonographic findings suggestive of obstruction, which, when interpreted in conjunction with the clinical history, physical examination, and hormonal profile, facilitated timely diagnosis and management. This facilitated timely clinical decision-making and subsequent sperm retrieval without unnecessary delays. This case underscores the intersection of pediatric urology and adult infertility, illustrating that even decades after a “successful” childhood urologic surgery, a history of childhood genitourinary surgery may occasionally become relevant during adult infertility evaluation. It also highlights the clinical value of POCUS in the andrology clinic as an extension of physical examination. Incorporating POCUS can significantly aid the differential diagnosis of azoospermia, guide appropriate management, and alleviate patient distress.

This case highlights the need for larger cohort studies to investigate the long-term fertility outcomes of men who underwent Cohen ureteral reimplantation in infancy. Prospective studies tracking fertility parameters in this population, coupled with detailed imaging assessments, could help quantify the risk of OA and elucidate the precise anatomical mechanisms of obstruction. Additionally, further research into standardized POCUS protocols for fertility specialists in azoospermia workup is warranted to enhance its widespread adoption and diagnostic accuracy.

## Data Availability

No datasets were generated or analysed during the current study.

## Abbreviations

OA: Obstructive Azoospermia
NOA: Nonobstructive Azoospermia
POCUS: Point–of–Care Ultrasonography
VUR: Vesicoureteral Reflux
FSH: Follicle–Stimulating Hormone
TESA: Testicular Sperm Aspiration
IVF: In Vitro Fertilization
ICSI: Intracytoplasmic Sperm Injection
PPV: Positive Predictive Value
NPV: Negative Predictive Value
TRUS: Transrectal Ultrasonography
